# Artificially Shortened Funis—with Rupture before Delivery

**Published:** 1847-04

**Authors:** Wilson Jewell


### MEDICAL EXAMINER

AND

### RECORD OF MEDICAL SCIENCE.

NEW SERIES.—No. XXVIII.—APRIL, 1847.

### ORIGINAL COMMUNICATIONS.

### *Artificially Shortened Funis—with Rupture before Delivery.*

Read before the Northern Medical Association of Philadelphia, January 21st, 1847. By Wilson Jewell, M. D.

Numerous causes have been assigned by obstetricians for protracted and difficult labour. Among others will be found, "a short funis," and "one or more coils of the cord around the neck of the child." The first of these may be viewed as a natural or an absolute, the latter, as an accidental or relative cause for delay in the birth. A cord short enough to retard delivery, could in no instance be coiled around the neck of the child, and a cord of a length sufficient to enable the child to pass through a loop once or oftener, could be no hindrance to the free exit of the fœtus without this occurrence, hence, these may be considered and treated of, as separate and distinct causes for tedious labour.

A shortened funis, either natural or accidental is so seldom the cause of tedious labour, or so rarely demands artificial aid in the progress of delivery, that some authors have not classed it among the causes for complicated labour, while others lay but slight stress upon it, deeming it of too little importance to require a special section for its consideration. Yet it does occur, though but rarely, and according to Moreau is one of the causes in the fœtus for rendering natural labour artificial—is an obstacle to parturition, exposing the mother to accidents, and endangering the life of the child.

Collins, in his practical treatise on midwifery, makes no allusion whatever to a naturally shortened funis, as though it never occurred, and has only a bare reference to an artificial shortening of the cord, under the head of natural labours, where he directs that the cord when around the neck should be brought over the head or passed back over the shoulders—for fear that the cord might act as a ligature, pressing on the neck of the child and checking the circulation—or injure the mother by dragging away the placenta, causing hemorrhage, or by inverting the uterus; but he gives no certain directions how to proceed in the event of not being able to remove the cord, either by passing it over the head or back over the shoulders. Nor can I find, that in the 16,654 births of which he gives the result, there is one instance recorded of shortened funis.

Baudelocque, of whom our American Baudelocque, the lamented Dewees, said he held himself indebted for nearly all he knew on midwifery—Baudelocque, whose genius and well tried experience none will doubt,—holds the following sentiment on this point of practice, which he places under the head of causes of preternatural labour.

"It is generally supposed that the cord by its shortness, or frequent windings on the child, opposes obstacles to delivering; such as keeping the head back, or if it allows it to advance during pain, it makes it recede immediately after; thus mistaking the reaction of the perineum and bones of the cranium, for the action of the cord. This will point out the futility of the advice of some authors who recommend the cutting of the cord, &c."

Dewees follows in this instance, very closely his master Baudelocque, but goes a little further. In his section on "Too short a cord," he says, "I shall not positively deny, that too short a cord, either artificial or natural will interrupt a natural labour, but I must say, I have never seen an instance; and also that I entertain strong doubts of its possibility."

Rigby, in his valuable work on obstetrics, gives practical directions in case of an accidental short cord, and its acting as a ligature on the neck of the child, and where it cannot be removed, to divide it—but adds, "we believe that this is rarely if ever necessary; for in proportion as the child advances, so does the fundus descend, and thus relieves, in some measure, the tension to which the cord is exposed." Under the head of difficult labours from faulty condition of the parts which belong to the child, he admits that "the umbilical cord may obstruct labour, by either being too short, or rendered so from being twisted round some part of the child," but on the very next page, as though he had forgotten his admission which I have just quoted, he says, "we quite agree with Professor Naegle, that unusual shortness of the cord can rarely if ever retards labour," and then again on the following page he adverts to the twisting of the cord around the neck, and adds, that it may not only retard labour, but destroy the child, by preventing the free return of blood from the head, either before the birth, or during the actual process of labour.

He also cites La Motte's and Burton's cases, where it became necessary in one case to cut the cord, which was done before the head was expelled from the os externum, in order to relieve the mother from danger and save the life of the child.

The cautious Blundell, who is certainly a very discriminating and safe practitioner, treats this whole subject very loosely. On page 111,* he makes mention of the cord being coiled round the neck six or seven times, and gives directions about removing it, but closes his remarks by saying, "a better method is to leave the cord around the neck, until the body be born, when it may be disentangled with facility." Under the variety of laborious labour, page 312, the same author doubts whether brevity of the cord gives rise to laborious labours, and thinks the shortness of the cord never makes itself felt, until after the head has escaped the vagina.

Ramsbotham in his practical work on midwifery, does not cite a case, in all his experience, of a short funis either natural or artificial, becoming the cause of protracted or dangerous labour involving either mother or child.

Lee, on the other hand, p. 240,* says that a short cord is the cause of delay, and cites Smellie's case, where the funis was three times around the neck, and where labour was protracted several days. He also says, unequivocally that from shortness of the umbilical cord without surrounding the neck of the child, it has been torn during the expulsion of the trunk and lower extremities. When the cord is around the neck of the child, he says the labour is sometimes protracted, and the head of the child retracted or drawn up after every pain, and that the cord has to be tied and divided before the child's body can be born.

Burns says, the cord may be on the stretch, but it never happens that it is torn, p. 79. vol. 2.† When the head is born with the cord around the neck he says, that some advise the cord to be cut, but his opinion is, that such a course "is rarely necessary, for the child may be born, though the cord remain about the neck."

The late Dr. James of this city, was of opinion, that retraction of the head was rarely caused by shortening of the cord or its being coiled around the neck—but was occasioned by rigidity or reaction of the external parts of the mother.

*Edition Select Medical Library.
†James' Burns, Edition, 1817.

Denman, p. 326, notices delay and difficulty, where the head has been expelled and the funis twisted round the neck, and he says, "lest the placenta should be separated, or the body of the child be hindered from advancing till it suffers detriment, or is brought into absolute danger," the cord must be brought over the head. If this cannot be done, slide the funis back over the shoulders; if either of these intentions cannot be accomplished without violence, they must be omitted, nevertheless the child will be usually expelled, if we wait for the return of a few pains, which we may safely do without any other inconvenience than some increased distention of the perineum. Where, however, the child is retarded a long time, the funis may be divided before it is expelled.

Professor Meigs, in his excellent manual, "The Philadelphia Practice of Midwifery," designed principally for students, but containing many choice reflections, gives similar directions as those authors already quoted, in cases where the cord is around the neck of the child—but says, if "the child seems to be detained by the tightness of the cord, as does rarely happen, or in danger from the compression of its jugular veins, the funis may be cut with the scissors and tied after the delivery."

The umbilical cord varies in length; while it is usually found about the length of the fœtus, say from eighteen to twenty inches, it has been found as long as eighty-four inches by Desormeaux, and the shortest one known, was only two inches in length. This occurred in the General Lying-in-Hospital in London, where after two or three violent pains, the child was suddenly expelled and the cord was found ruptured two inches from its navel. The other end of the funis was sought for, but could not be found; on removing the placenta from the uterus, it was discovered that the cord had been ruptured at its very insertion.

The shortening of the umbilical cord, by being coiled around the neck of the child, is of frequent occurrence, and according to some authors, takes place as often as once in five or six cases. I agree in opinion with those writers whom I have quoted, that the cases of difficulty arising from such a shortening are rare. But that they do occur, that they may cause delay, retard delivery, endanger the life of the child, and that the cord may be ruptured before the delivery of the body of the child, I would present the following case in point, from my memorandum of cases.

On the twentieth day of August, 1846, I was sent for about 12, M., to visit Mrs. R. in labour with her fifth child. As her previous accouchement had been remarkably quick, and as I had been informed, so were her former ones, I made all haste and was soon at her bedside, where I found her in strong pains. An examination per vaginam, led at once to the opinion that I was "just in time" to receive the child, as the head was pressing forcibly during the pain against the perineum. I was, however, doomed to experience disappointment in my sanguine hope of a speedy delivery and a short detention. As the pain subsided, the head receded quite high up, and remained there, until the following pain brought it down once more to the "crowning" point of labour. Again and again, for many successive pains, I was obliged to contend with this alternate advancing and receding of the head, until the clock struck two, and until my patience was wearied, and my patient and her friends wondered at the delay.

Although unable to diagnosticate by the touch without the introduction of the hand, yet I surmised and even communicated my suspicions as to the cause of the impediment, to be, either a natural or artificial shortening of the funis. I was led to this opinion, because there was a normal conformation of the pelvis of the mother and the fœtal head of the child, because the soft parts were fully and sufficiently relaxed for every necessary purpose, because the uterine contractions were vigorous and frequent, and because the head was in the first position of Baudelocque, being the most favorable for the termination of labour without the interposition of art, unless there existed some mal-conformation of the body of the fœtus, or, as I suspected in the present case, either an artificial or natural shortening of the umbilical cord.

For more than two hours I waited and watched anxiously the result. At length a powerful and continued effort of the womb expelled the head of the child beyond the vulva, but when the uterine action had ceased, the head remained firmly wedged against the os externum.

The appearance of the child at this stage of the birth, indicated immediate strangulation, unless relief could be afforded, as I now discovered by passing my finger around the neck, that it was closely compressed by being encircled with two turns of the cord, which by this arrangement, was artificially shortened. The face was now assuming a very dark purple colour from the accumulation of blood in the return vessels, in consequence of the obstruction of the jugular veins. There was no time to lose. Accordingly, I made an effort to remove the cord by bringing down a loop and passing it over the head, but it was drawn so tight that I found it difficult even to insert my finger beneath it, much less to bring down a portion and slip it over the head or pass it back over the shoulders, as various authors direct.

Thus situated, I was in momentary fear of witnessing those spasmodic jerks, which are so clearly indicative of the death of the fœtus, when another pain returned. At this crisis, I determined to pass my finger beneath that portion of the cord which was coiled around the neck, not only to prevent an increase of pressure on the circulation of the jugular vessels, but to separate the cord and thus relieve the extreme danger, when suddenly the cord gave way, and almost at the same moment of time the child was precipitated into the world and lay at the breech of the mother. The bleeding from the ruptured umbilical extremity of the funis soon relieved the apoplectic symptoms. I put a ligature on the umbilical extremity of the cord, which, singular to relate, was ruptured about two inches from the navel. In a very few minutes the child breathed and cried freely, and I handed to the nurse a fine healthy boy.

The placenta came away in season, and from that hour the mother and child did well.

There are two points in the above case of "artificially shortened funis with rupture before delivery," which may afford us subjects for practical reflection.

1. Can a shortened funis obstruct delivery?

2. Would it endanger the life of the mother or child?

All authors to whom I have had access, agree that one or more coils of the umbilical cord around the neck of the fœtus is a common occurrence, happening as often as once in every six or seven labours; but that it ever changes a natural into a preternatural labour, they are not so well agreed.

Baudelocque, Dewees, James, Burns and Blundell, do not believe that a short cord ever protracts or retards labour, while on the other hand, Smellie, Rigby, Lee, Moreau, Denman and Meigs are of opinion that it may produce delay, though rarely, and each of them give some directions how to proceed when it does occur.

I fully agree in opinion with these last named gentlemen, and although my case was not protracted for several days as was that of Smellie's, still it does not change the principle, nor my opinion, that the case of Mrs. R. was hindered for two hours, and perhaps longer, by an artificially shortened funis.

Would a shortened funis endanger the life of the mother or child?

Here I have not the least hesitation in pronouncing judgment in the affirmative, and am supported in my opinion by Baudelocque, Lee, Moreau and others.

In the expulsion of the fœtus, the violence of the dragging pains may tear the placenta from the womb, and expose the mother to a destructive flooding, or produce an inversion of that organ, or the pressure of the cord around the neck of the child may retard the free circulation of the blood in the brain, producing asphyxia, apoplexy and death, or should the cord rupture and assistance not be at hand, the child might perish from the loss of blood. On this point there can be but little difference of opinion. Almost every author I have consulted, gives short directions for the removal of the cord from the neck of the child, and several of them recommend the cutting of it before the body shall be delivered, in order to protect its life.

Who would be willing to follow the recommendation of Blundell, and believe with him that "a better method" is to leave the cord around the neck until the body of the child is born, rather than remove it or cut it, when it was evident from the livid suffusion of the countenance of the half-born fœtus, that the circulation was impeded and death threatening? It is in such a case that procrastination would be attended with danger. It is here, that a "meddlesome midwifery," to employ in part the language of this fanciful writer, would be proper, rather than trust the case in the hands of that "excellent accoucheur in natural labour—Nature, the mother of us all."

The case I have presented is one of interest, and not only rare in its occurrence, but still more novel in its character; for as far as my researches have extended, I find no such instance on record. Baudelocque believes that it is possible for a cord, if short, to be ruptured before delivery; and Rigby says it has taken place, and he furnishes a reference in the London Med. and Surg. Journ. for May, 1835, p. 426, of a case which happened in the London General Hospital—but it was one of natural shortening of the cord—which was only two inches in length, and was ruptured before the child could be born. La Motte's cases and Burton's cases, as cited by Rigby, were artificial shortenings, but in neither of these did a rupture take place spontaneously before delivery. Burton ruptured both of his, and La Motte, who had three cases, cut one with his scissors, dragged another away with the placenta, and the third was delivered with little else than force.

